# The impact of the COVID-19 pandemic on tuberculosis treatment outcomes in 49 high burden countries

**DOI:** 10.1186/s12916-024-03532-7

**Published:** 2024-07-29

**Authors:** Vester Gunsaru, Marc Y. R. Henrion, C. Finn McQuaid

**Affiliations:** 1 Malawi Liverpool Wellcome Programme, Blantyre, Malawi; 2 Liverpool School of Tropical Medicine, Liverpool, UK; 3 TB Modelling Group, TB Centre and Centre for Mathematical Modelling of Infectious Diseases, Department of Infectious Disease Epidemiology, Faculty of Epidemiology and Population Health, London School of Hygiene & Tropical Medicine, London, UK

**Keywords:** Tuberculosis, COVID-19, Treatment, Outcomes

## Abstract

**Background:**

The COVID-19 pandemic disrupted tuberculosis (TB) health services, including treatment support and access to drugs, as patients were not able to access health facilities. While the effect of this disruption on treatment outcomes has been studied in isolated treatment centres, cities and provinces, the impact of the pandemic on TB treatment outcomes at a country and regional level has not been evaluated.

**Methods:**

We used treatment outcomes for new and relapse TB cases reported to the World Health Organization (WHO) from 49 high TB, TB/HIV and drug-resistant TB burden countries from 2012 to 2019. We developed multinomial logistic regression models for trends in TB treatment success, failure, death and loss to follow up. We predicted TB treatment outcomes for 2020 and 2021, comparing these to observations, by computing ratios between observed and predicted probabilities. We aggregated these risk ratios (RR) for six WHO-defined regions using random-effects meta-analysis.

**Results:**

Across 49 countries and four TB treatment outcomes, 17 (out of 196) country-outcome pairs in 2020 and 21 in 2021 had evidence of systematic differences between observed and predicted TB treatment outcome probabilities. Regionally, only four (out of 24) region-outcome pairs had evidence of systematic differences in 2020 and four in 2021, where the European region accounted for four of these in total. Globally, there was evidence of systematic differences in treatment failure in both 2020 (RR: 1.14, 95%CI: 1.01–1.28, *p* = 0.0381) and 2021 (RR: 1.36, 95%CI: 1.03–1.78, *p* = 0.0277), deaths in 2020 (RR: 1.08, 95%CI: 1.03–1.13, *p* = 0.0010) and losses to follow up in 2020 (RR: 0.91, 95%CI: 0.86–0.97, *p* = 0.0059).

**Conclusions:**

While for some countries and regions there were significant differences between observed and predicted treatment outcomes probabilities, there was insufficient evidence globally to identify systematic differences between observed and expected TB treatment outcome probabilities because of COVID-19-associated disruptions in general. However, larger numbers of treatment failures and deaths on treatment than expected were observed globally, suggesting a need for further investigation.

**Supplementary Information:**

The online version contains supplementary material available at 10.1186/s12916-024-03532-7.

## Background

The COVID-19 pandemic resulted in major disruptions to tuberculosis (TB) diagnosis and prevention [1–3]. In particular, a significant decrease was seen globally in the number of people with TB who were able to access diagnosis and were reported to the health system, with 1.3 million fewer individuals notified in 2020 than 2019 [1]. Over the same period 700,000 fewer individuals accessed TB preventive treatment [1], primarily people living with HIV. In 2022, the world started recovering from these disruptions, with 1.1 million more notified cases than 2021, and 1.7 million more notified cases than 2020 [1].

At the same time, the pandemic disrupted access to TB treatment. Patients experienced delays to treatment initiation, medication stock-outs and lack of access, and disruptions to treatment support [4], with potential interruptions to treatment adherence as a consequence. Poor adherence to TB treatment itself, whether starting treatment late, ending treatment early, or intermittently missing doses [5], can lead to poor treatment outcomes, including disease relapse and drug-resistance [6, 7]. Consequently, these interruptions to TB treatment could potentially have major repercussions for TB treatment outcomes, potentially reducing treatment success, and increasing treatment failure, loss to follow up and death on treatment.

Previous studies in high TB burden settings indicate a mixture of evidence for this. Smaller studies in individual treatment centres, cities and provinces in China [8], Ethiopia [9, 10], Zimbabwe [11, 12], Eswatini [13], India [14–16], Kazakhstan [17], Uganda [18] and Mexico [19] suggest that treatment outcomes may have deteriorated. However, a handful of other small studies in Kenya [12, 20], Malawi [12, 21], Sierra Leone [22] and Indonesia [23], as well as much larger studies in Lesotho [24], Indonesia [25], Peru [26], Brazil [27] and Vietnam [28], do not appear to have experienced this deterioration. More recent evidence suggests that at a global level treatment outcomes do not appear to have been affected by the pandemic [1]. However, this may mask significant variations at a country level as well as sub-nationally.

Here we investigated whether disruptions to TB treatment resulted in worsening treatment outcomes in 49 high TB burden countries and globally. We hypothesized that COVID-19-associated disruptions would lead to a decrease in treatment success and an increase in treatment failure, relapse and death on treatment, as compared to expected values.

## Methods

### Data

We used country level treatment outcome data for new and relapse drug-susceptible TB cases reported to the World Health Organization (WHO) by individual WHO region countries. These were data recorded on TB treatment success, failure, death and loss to follow up for the years 2012 – 2021 [29]. We focused on WHO high TB, TB/HIV and multidrug/rifampicin-resistant TB (MDR/RRTB) burden countries [30].

### Analysis

We fitted multinomial logistic regression (MLR) models to data from 2012–2019 to estimate the expected probabilities of TB outcomes in 2020 and 2021 for each country. The MLR model included time $t$ ($t=$ *2012, 2013, …,2019*) as a predictor variable. We used natural cubic regression splines to model the non-linear relationship between TB treatment outcomes and time with $K$ knots. Natural splines were used due to having linear functions in the boundary knots tails which provided more stable model fits. The probability of each level *j* of $Y,$ the TB treatment outcome for country *c* was then given by:

$$\text{Pr}\left({Y}_{cjt}\right) =\text{Pr}\left({Y}_{c}=j \right| t) = \frac{{e}^{{\beta }_{ocj} +{ \beta }_{1cj}t + \sum_{k=1}^{K-2}{\beta }_{kcj}{\left(t-{\tau }_{k}\right)}_{*}^{3}}}{1 + \sum_{l=1}^{J-1}{e}^{{\beta }_{0cl} +{ \beta }_{1cl}t + \sum_{k=1}^{K-2}{\beta }_{kcl}{\left(t-{\tau }_{k}\right)}_{*}^{3}}}$$

for j = 1, …, J-1, and for the last level J:

$$\text{Pr}\left({Y}_{cJt}\right) =\text{Pr}\left({Y}_{c}=J \right|t) = \frac{1}{1 + \sum_{l=1}^{J-1}{e}^{{\beta }_{0cl} +{ \beta }_{1cl}t + \sum_{k=1}^{K-2}{\beta }_{kcl}{\left(t-{\tau }_{k}\right)}_{*}^{3}}}$$

The natural logarithm of the probability then gives:

$$\text{ln}\left(\frac{\text{Pr}(Y_{cjt})}{\text{Pr}(Y_{cJt})}\right)=\beta_{ocj}+\beta_{1cj}t+{\textstyle\sum_{k=1}^{K-2}}\;\beta_{kcj}\left(t-\tau_k\right)_\ast^3,j=1,\;\dots\;,J$$

Where ${\tau }_{k}$ are *t* values at knot $k$ and for $k$ = 1, …, $K-2$,

$${\left(t- {\tau }_{k}\right)}_{*}^{3}= {\left(t- {\tau }_{k}\right)}_{+}^{3}- {\left(t- {\tau }_{K-1}\right)}_{+}^{3}\frac{{\tau }_{K}- {\tau }_{k}}{{\tau }_{K}- {\tau }_{K-1}}+ {\left(t- {\tau }_{K}\right)}_{+}^{3}\frac{{\tau }_{K-1}- {\tau }_{k}}{{\tau }_{K}- {\tau }_{K-1}},$$

$$\begin{aligned}{\left(t-\tau_k\right)}_{+}=\big\{ \big\{\begin{array}{l} ct-\tau_k:if\,t-\tau_k>0\\0:if\,t-\tau_k\leq0\end{array} \end{aligned}$$

Once the models were fitted, we computed the model-predicted treatment outcome probabilities for 2020 and 2021 and compared these to the observed outcome proportions for 2020 and 2021 for each country, by computing the ratio between the observed and the expected values. Bootstrap sampling was used to derive confidence intervals for the estimated outcome probabilities, and, in turn, the ratios between observed and expected proportions and their corresponding *p*-values.

To pool the effect sizes, and given the diverse set of countries in our dataset, we conducted random-effects meta-analysis of the ratios between observed and predicted outcome probabilities, overall and by WHO region. We estimated the percentage of the variation across studies not due to sampling error using the *I*^*2*^ statistic. Restricted maximum-likelihood estimator was used to estimate between-country variance. All analyses were done in the R (version 4.3.1) environment for statistical computing [31–44].

We considered there to be evidence of systematic differences between estimated and observed treatment success probabilities if the *p*-value for the ratio was < 0.05.

## Results

We included data from 49 high TB, TB/HIV and MDR/RRTB burden countries that were reported to the WHO from 2012–2021 (Kazakhstan and Uzbekistan did not have data for 2021). The 49 countries included 24 from the WHO African region, nine from the European region, seven from South-East Asia region, two from the Region of the Americas, five from Western Pacific region and two from the Eastern Mediterranean region. The observed probability of TB treatment success aggregated across all countries indicated a general increase from 2012 to 2021. A similar general decrease was seen for treatment failure and loss -to -follow up. Observed proportions of deaths fluctuated over time (Table 1).

**Table 1:** Probabilities of TB treatment outcomes by year (2012–2021) aggregated across 49 high TB, TB/HIV and MDR/RR-TB burden countries, as a weighted average of country-specific outcomes

	**2012**	**2013**	**2014**	**2015**	**2016**	**2017**	**2018**	**2019**	**2020**	**2021**
Success	0.9043	0.9063	0.9072	0.9102	0.9038	0.9110	0.9061	0.9111	0.9128	0.9176
Failure	0.0118	0.0111	0.0096	0.0092	0.0131	0.0092	0.0076	0.0073	0.0072	0.0074
Death	0.0398	0.0391	0.0392	0.0391	0.0407	0.0379	0.0369	0.0387	0.0420	0.0394
Loss to follow up	0.0441	0.0436	0.0439	0.0414	0.0424	0.0419	0.0493	0.0428	0.0380	0.0356

**Fig. 1:**
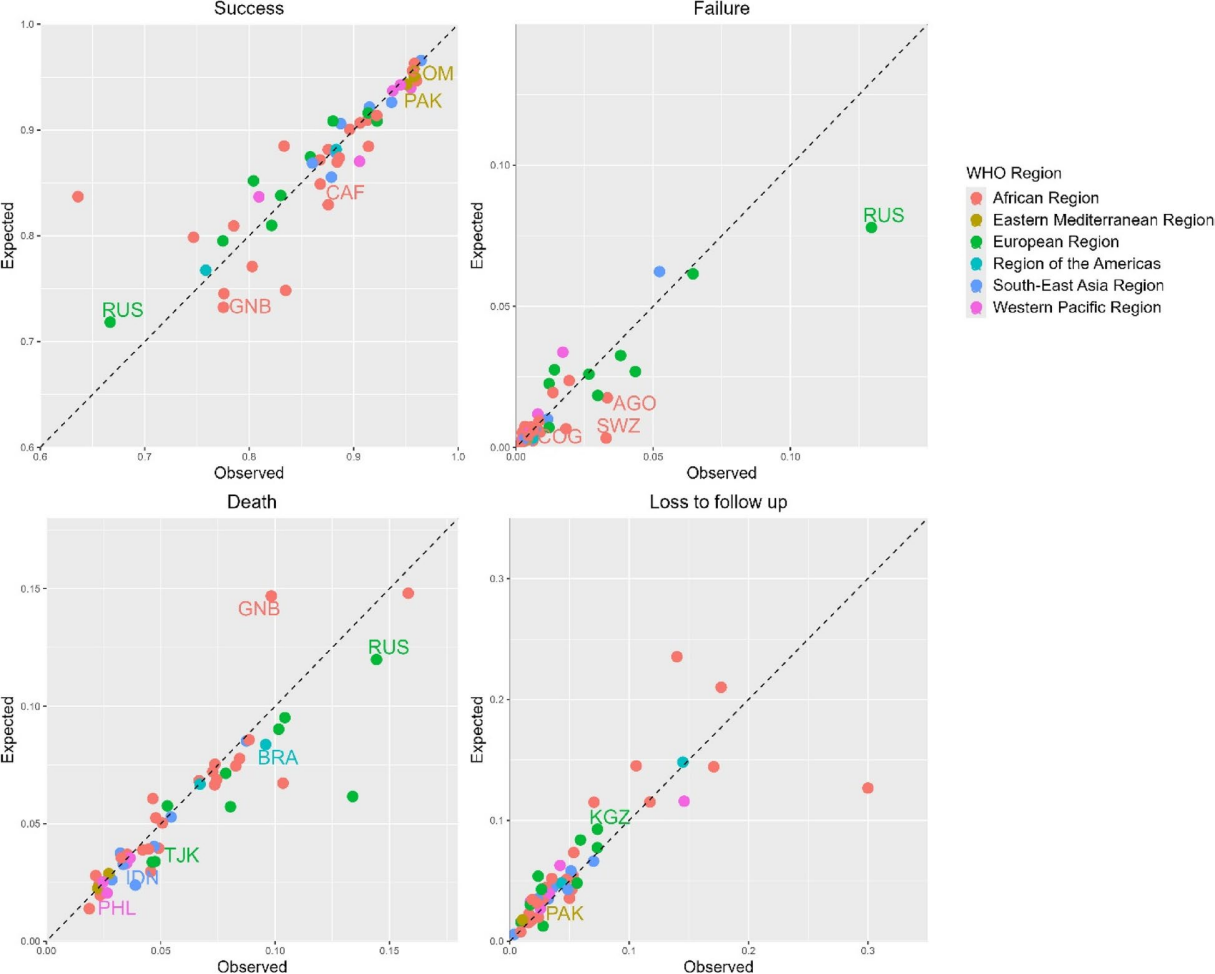
Observed and expected tuberculosis treatment outcome probabilities for 49 high TB, TB/HIV and MDR/RR-TB burden countries in 2020. Labelled are those with statistically significant differences: AGO – Angola, BRA – Brazil, CAF – Central African Republic, COG – Congo, GNB – Guinea-Bissau, IDN – Indonesia, KGZ – Kyrgyzstan, PAK – Pakistan, PHL – Philippines, SOM – Somalia, SWZ- Eswatini, TJK—Tajikistan and RUS – the Russian Federation

Comparing the observed and estimated TB treatment success from the MLR models in 2020 (Fig. 1), the Central African Republic (1.06, 95% CI: 1.01–1.39, *p* = 0.0259), Guinea-Bissau (1.06, 95% CI: 1.01–1.22, *p* = 0.0339), Pakistan (1.01, 95% CI: 1.00–1.06, *p* = 0.0218), the Russian Federation (0.93, 95% CI: 0.87–0.98, *p* = 0.0338) and Somalia (1.01, 95% CI: 1.00–1.05, *p* = 0.0143) had statistically significant ratios, i.e. there were more observed successes than expected in all countries except in the Russian Federation where there were fewer. For 2021 (Additional file 1: Fig. S1), Bangladesh (1.01, 95% CI: 1.00–1.02, *p* = 0.0468), the Central African Republic (1.06, 95% CI: 1.00–1.71, *p* = 0.0434), Pakistan (1.02, 95% CI: 1.01–1.11, *p* = 0.0116), the Philippines (0.98, 95% CI: 0.96–0.99, *p* = 0.0339) and Zimbabwe (1.03, 95% CI: 1.00–1.35, *p* = 0.0240) had statistically significant ratios, i.e. there were more observed successes than expected in all other countries except in the Philippines where there were fewer.

Comparing observed and estimated TB treatment failure in 2020, statistically significant ratios were observed in Angola (1.90, 95% CI: 1.06–7.19, *p* = 0.0450), Congo (2.59, 95% CI: 1.26–55.18, *p* = 0.0232), Eswatini (10.03, 95% CI: 3.62–40.54, *p* = 0.0052) and the Russian Federation (1.66, 95% CI: 1.15–2.37, *p* = 0.0229), i.e. there were more observed failures than expected in these countries. For 2021, Congo (5.54, 95% CI: 1.91–651.18, *p* = 0.0137), Nigeria (16.68, 95% CI: 6.43–24.13, *p* = 0.0015), the Russian Federation (1.70, 95% CI: 1.05–3.01, *p* = 0.0352) and Viet Nam (2.03, 95% CI: 1.21–32.49, *p* = 0.0249) had statistically significant ratios in treatment failure, i.e. there were more observed failures than expected in these countries.

Comparing observed and expected TB deaths in 2020, statistically significant ratios were observed in Brazil (1.15, 95% CI: 1.08–1.30, *p* = 0.0180), Guinea-Bissau (0.67, 95% CI: 0.45–0.96, *p* = 0.0458), Indonesia (1.63, 95% CI: 1.36–2.44, *p* = 0.0022), Philippines (1.29, 95% CI: 1.08–1.66, *p* = 0.0337), the Russian Federation (1.20, 95% CI: 1.07–1.29, *p* = 0.0140) and Tajikistan (1.40, 95%CI: 1.04-1.77, *p* = 0.0159), i.e. there were more observed deaths than expected in these countries except in Guinea Bissau where there were fewer. For 2021, Brazil (1.26, 95% CI: 1.13–1.51, *p* = 0.0153), Eswatini (2.30, 95% CI: 1.05–6.18, *p* = 0.0467), Guinea-Bissau (0.39, 95% CI: 0.24–0.71, *p* = 0.0208), Indonesia (1.70, 95% CI: 1.25–3.14, *p* = 0.0064), Mozambique (1.71, 95% CI: 1.04-2.30, *p* = 0.0422), Nigeria (0.09, 95% CI: 0.03–0.13, *p* = 0.0017), the Russian Federation (1.24, 95% CI: 1.06–1.37, *p* = 0.0188) and Ukraine (1.27, 95% CI: 1.03–1.72, *p* = 0.0315) had statistically significant ratios in TB deaths. There were more observed deaths than expected in Brazil, Indonesia, Mozambique, the Russian Federation and Ukraine, and less observed deaths than expected in Guinea-Bissau and Nigeria.

Comparing observed and expected loss to follow up in 2020, statistically significant ratios were observed in Kyrgyzstan (0.79, 95% CI: 0.45–0.93, *p* = 0.0322) and Pakistan (0.72, 95% CI: 0.48–0.94, *p* = 0.0413), i.e. there were less observed losses to follow up than expected. For 2021, Belarus (5.76, 95% CI: 1.25–17.93, *p* = 0.0348), Pakistan (0.60, 95% CI: 0.35–0.96, *p* = 0.0434), the Philippines (1.45, 95% CI: 1.12–2.48, *p* = 0.0307) and the Russian Federation (0.52, 95% CI: 0.46-0.96, *p* = 0.0432) had statistically significant ratios in losses to follow up. There were more observed losses to follow up than expected in Belarus and the Philippines, and less observed losses to follow up than expected in Pakistan and the Russian Federation.

See Additional file 1: Tables S1, S2, S3, S4, S5, S6, S7, S8, S9, S10, S11, S12, S13, S14 for further details on differences between observed and expected TB treatment outcomes in 2020 and 2021.

### Meta-analyses

**Fig. 2:**
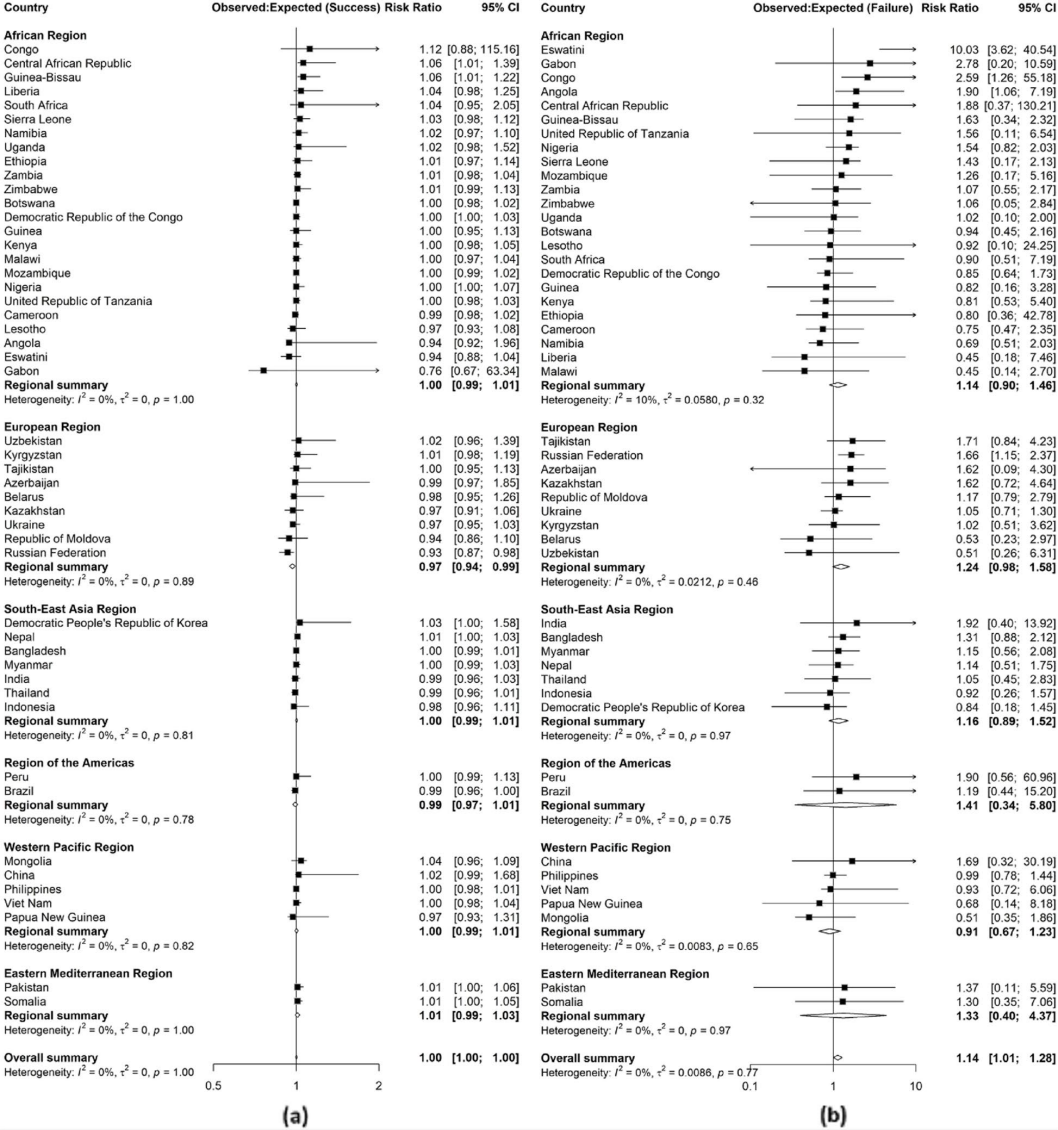
Random effect meta-analyses forest plots highlighting ratios between observed and expected proportions for tuberculosis treatment success (**a**) and failure (**b**) in 2020 for 49 high TB, TB/HIV and drug resistant TB burden countries by WHO region. Greater than one ratio imply that the observed proportions were more than expected, and less than one ratio imply that the observed proportions were less than the expected

Pooled effects for TB treatment success at WHO regional level showed evidence of systematic differences between observed and expected probabilities in 2020 (Fig. 2a) in the European Region (0.97, 95% CI: 0.94-0.99, *p* = 0.0140, $I^{2}$ =0%), and in 2021 (Additional file 1: Fig. S2a) in the European Region (0.94, 95% CI: 0.90-0.99, *p* = 0.0103, $I^{2}$ = 0%) and the Western Pacific Region (0.98, 95% CI: 0.97-1.00, *p* = 0.0153, $I^{2}$ = 0%). However, there was no evidence of systematic differences globally both in 2020 and 2021.

There was no evidence of systematic differences for treatment failure in any of the regions but there was evidence of systematic differences globally in both 2020 (1.14, 95% CI: 1.01-1.28, *p* = 0.0381, $I^{2}$ = 0%) (Fig. 2b) and 2021 (1.36, 95% CI: 1.03-1.78, *p* = 0.0277, $I^{2}$ = 43.7%) (Additional file 1: Fig. S2b).

**Fig. 3:**
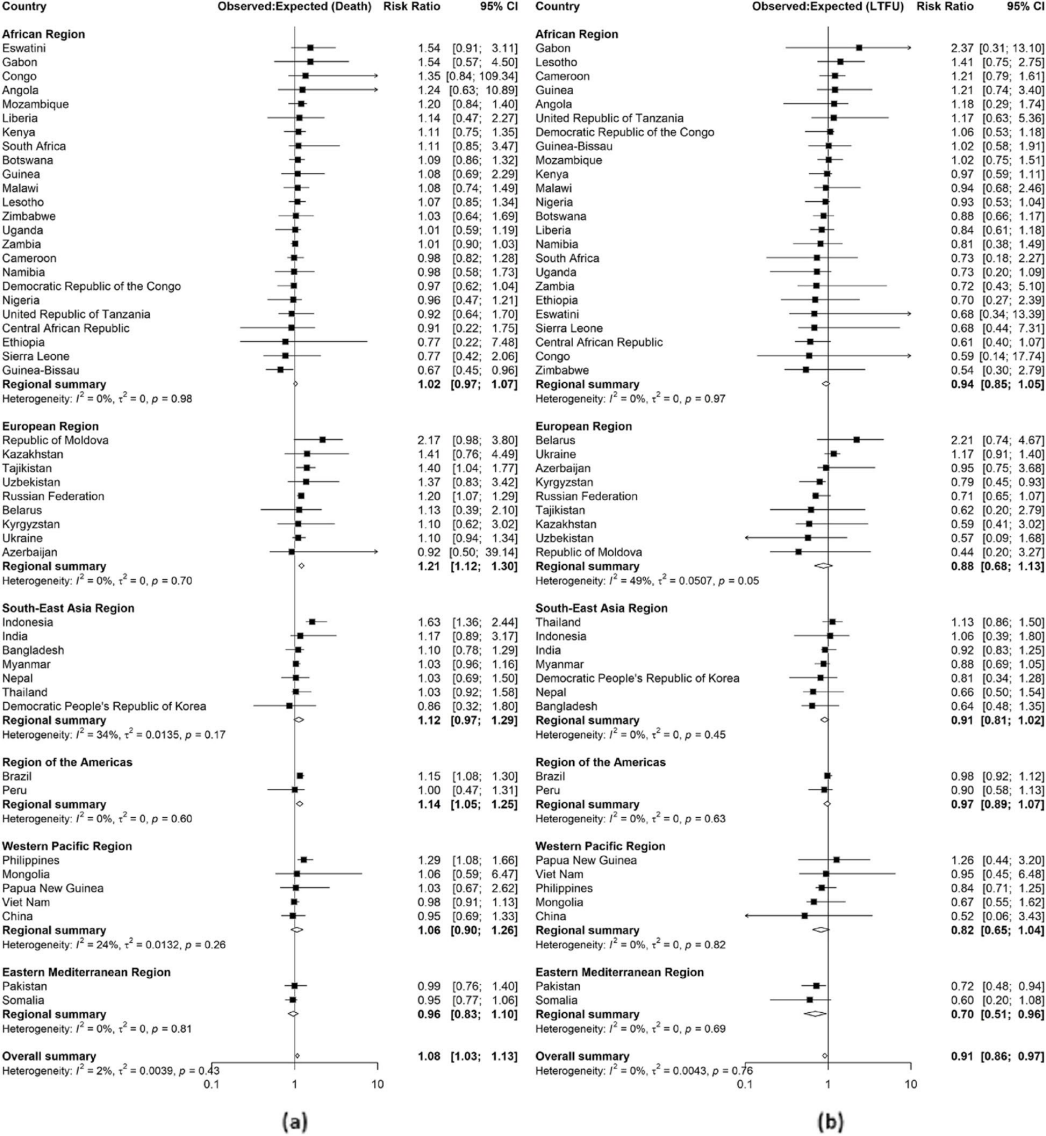
Random effect meta-analyses forest plots highlighting ratios between observed and expected proportions for tuberculosis treatment death (**a**) and loss to follow up (LTFU) (**b**) in 2020 for 49 high TB, TB/HIV and drug resistant TB burden countries by WHO region. Greater than one ratio imply that the observed proportions were more than expected, and less than one ratio imply that the observed proportions were less than the expected

There was evidence of systematic differences for TB deaths in 2020 (Fig. 3a) in the European Region (1.21, 95% CI: 1.12-1.30, *p* = 1.75e-06, $I^{2}$ = 0%) and the Region of the Americas (1.14, 95% CI: 1.05-1.25, *p* = 0.0036, $I^{2}$ = 0%), and in 2021 (Additional file 1: Fig. S3a) in the European Region (1.26, 95% CI: 1.13-1.40, *p* = 3.07e-05, $I^{2}$ = 0%) and the Region of the Americas (1.24, 95% CI: 1.08-1.43, *p* = 0.0027, $I^{2}$ = 0%). There was also evidence of systematic differences globally (1.08, 95% CI: 1.03-1.13, *p* = 0.0010, $I^{2}$ = 2.0%) in 2020.

Lastly, there was evidence of systematic differences for loss to follow up in 2020 (Fig. 3b) in the Eastern Mediterranean Region (0.70, 95% CI: 0.51-0.96, *p* = 0.0265, $I^{2}$ = 0%) and globally (0.91, 95% CI: 0.86-0.97, *p* = 0.0059, $I^{2}$ = 0%). There was no evidence of systematic differences in 2021 (Additional file 1: Fig. S3b).

## Discussion

Across 49 countries and two years, statistically significantly more treatment successes were observed than expected in eight countries and fewer treatment successes were observed than expected in two countries. Significantly more treatment failures than expected were observed in eight countries, while more deaths than expected were observed in 11 countries and less deaths than expected were observed in three countries across the two years. Statistically significantly more losses to follow up than expected were observed in two countries and less losses to follow up than expected were observed in four countries across 2020 and 2021. Regionally, only four (out of 24) region-outcome pairs had evidence of systematic differences in 2020 and four in 2021, where the European region accounted for four of these in total. Globally, there was evidence of systematic differences in the failure outcome in both 2020 and 2021, death outcome in 2020 and loss to follow up in 2020.

Our results are limited by the fact that treatment outcomes (where treatment success is defined as a combination of cure and treatment completion [29]), are comparatively blunt indicators. For example, it may be that patients were considered to have completed treatment six months after enrollment, without receiving a negative bacteriological test or ingesting the recommended number of drugs. An increase in the proportion of treatment success that was a result of treatment completion may mask a decrease in patients who were actually cured of TB. The significant observed reduction in TB diagnosis [1] could also have resulted in those who were at an increased risk of poor treatment outcomes not accessing diagnosis, such that the populations we compare before and during the pandemic may be significantly different. While studies have identified groups with differential treatment outcomes potentially at an increased risk of missed or delayed diagnosis [45], other risk factors not included in routine reporting could also play a role here. Indeed, due to the structure of reporting systems there is a possibility that any increase in death seen in our results is a consequence of an increase in death due to other causes (in particular, COVID-19), rather than TB. This is particularly likely in countries where the population with TB is at an increased risk, such as older populations or populations with multiple comorbidities. These outcomes also represent an aggregation of all patients in a country who received treatment across an entire year. It is not possible to identify whether those who did so during stringent lockdowns may have been more affected than those who received treatment during periods where lockdowns were less stringent or non-existent. Similarly, those treated in 2021 may represent individuals who experienced a delayed diagnosis, and potentially therefore more severe disease, in 2020 as a result of COVID-19-assocaited disruptions. This increase in disease severity could have affected treatment outcomes in 2021, and may still for future years, however it is not possible to disaggregate these results here. It is also not possible to identify variation at a more granular geographic scale, where previous centre-, city-, and province-specific studies suggest that this could be important. At the same time, there are limitations in the data available for analysis, where we considered just one predictor (time) with no other available factors that may have changed during the study period and that may have played a role in TB treatment outcomes across different countries. Lastly, our analysis focuses on drug-susceptible TB; given the differences in care between drug susceptible and MDR/RR-TB, such as drug-susceptibility monitoring and extended duration of treatment, it would be inadvisable to extrapolate our findings to MDR/RR-TB.

Our results are in line with a number of other country-level studies in finding that, unlike widespread reductions in TB diagnosis and prevention, at a country level TB treatment outcomes do not appear to have been noticeably affected by disruptions associated with the pandemic. In particular, treatment success remained high, and loss to follow up and relapse were not widely affected. It is likely that the pandemic saw an increase in risk factors associated with poor TB treatment outcomes, such as untreated HIV [1], undernourishment [46], poverty [47], alcohol use [48] and many others [49]. In this context, given the individual-level consequences of poor treatment outcomes including TB relapse, lung damage and death, this achievement should be celebrated. A widespread switch to digital treatment adherence technologies to support treatment [50], as well as significant efforts on the part of healthcare workers to ensure patients were able to access medicines [51], may have helped to reduce the effect of these disruptions, potentially including a reduction in loss to follow up.

However, where we did observe a change in TB treatment outcomes, this was broadly a decrease in treatment success and an increase in poor treatment outcomes. A small number of countries, in particular Eswatini, Guinea-Bissau, Nigeria, Pakistan, Philippines, and the Russian Federation, also appeared to be more affected than others with multiple indicators affected, warranting further investigation. For example, in Nigeria this may represent changes due a recent concerted effort to increase case finding during the study period, rather than as a result of COVID-19-assocaited disruptions. Most strikingly, evidence from our analysis that TB treatment failure and deaths on treatment may have increased in 2020 requires further study, although a relatively small effect size suggests that there may not be cause for serious concern. Overall, therefore, although we found limited evidence at a country level that TB treatment outcomes worsened as a result of the pandemic, we did find some evidence at a global level that certain poor outcomes increased. As a result, fears that potentially thousands more people treated for TB might experience long-term consequences as a result of pandemic-associated disruptions appear to warrant further investigation.

## Conclusions

Our study found limited evidence of COVID-19 associated disruptions on TB treatment outcomes in high burden countries and globally. Some countries registered fewer treatment successes than expected and more failures, deaths and losses to follow up than expected. Globally, however, there were more treatment failures and deaths than expected in 2020, but less losses to follow up. Given these findings, in a future pandemic, it is crucial that TB treatment outcomes are closely monitored so that, where necessary, TB treatment initiatives and campaigns may be intensified to achieve better outcomes.

### Supplementary Information

Additional file 1: Figures S1-S3 and Tables S1-S14. Fig. S1 – Country specific observed and expected tuberculosis treatment probabilities in 2021. Fig. S2 – Meta-analyses forest plots for successes and failures in 2021. Fig. S3 – Meta analyses forest plots for deaths and losses to follow up in 2021. Table S1 – WHO region specific observed and expected tuberculosis treatment outcomes in 2020. Table S2 – WHO region specific observed and expected tuberculosis treatment outcomes in 2021. Table S3 – Country specific observed and expected tuberculosis treatment outcomes for the African region in 2020. Table S4 – Country specific observed and expected tuberculosis treatment outcomes for the European region in 2020. Table S5 – Country specific observed and expected tuberculosis treatment outcomes for the South-East Asia region in 2020. Table S6 – Country specific observed and expected tuberculosis treatment outcomes for the region of the Americas in 2020. Table S7 – Country specific observed and expected tuberculosis treatment outcomes for the Western Pacific region in 2020. Table S8 – Country specific observed and expected tuberculosis treatment outcomes for the Eastern Mediterranean region in 2020. Table S9 – Country specific observed and expected tuberculosis treatment outcomes for the African region in 2021. Table S10 – Country specific observed and expected tuberculosis treatment outcomes for the European region in 2021. Table S11 – Country specific observed and expected tuberculosis treatment outcomes for the South-East Asia region in 2021. Table S12 – Country specific observed and expected tuberculosis treatment outcomes for the region of the Americas in 2021. Table S13 – Country specific observed and expected tuberculosis treatment outcomes for the Western Pacific region in 2021. Table S14 – Country specific observed and expected tuberculosis treatment outcomes for the Eastern Mediterranean region in 2021.

## Data Availability

All data generated or analysed during this study are included in this published article and its supplementary files or through the World Health Organization repository: https://www.who.int/tb/country/data/download/en/. The analysis code can be accessed via https://github.com/Vester123/TB-COVID-19-Manuscript.

## Abbreviations

CI: 95% Confidence interval
HIV: Human immunodeficiency virus
MDR/RR-TB: Multidrug- or rifampicin-resistant tuberculosis
MLR: Multinomial logistic regression
TB: Tuberculosis
WHO: World Health Organization
