# Correction: The impact of the COVID-19 pandemic on tuberculosis treatment outcomes in 49 high burden countries

**DOI:** 10.1186/s12916-024-03551-4

**Published:** 2024-08-02

**Authors:** Vester Gunsaru, Marc Y. R. Henrion, C. Finn McQuaid

**Affiliations:** 1 Malawi Liverpool Wellcome Programme, Blantyre, Malawi; 2 Liverpool School of Tropical Medicine, Liverpool, UK; 3 TB Modelling Group, TB Centre and Centre for Mathematical Modelling of Infectious Diseases, Department of Infectious Disease Epidemiology, Faculty of Epidemiology and Population Health, London School of Hygiene & Tropical Medicine, London, UK

**Correction** **:** **BMC Med 22, 312 (2024)**

**https://doi.org/10.1186/s12916-024-03532-7**

The original article [1] contains an error in the lower-rightmost equation in the lower-right hand corner of Page 2 of the PDF.

The correct presentation of the equation is as shown ahead:

$${\left(t-\tau_k\right)}_+=\left\{\begin{array}{cc}t-\tau_{k\;}&:\;if\;t-\tau_k>0\\0&:\;if\;t-\tau_k\leq0\end{array}\right.$$
