# Supplementary material for: The impact of the COVID-19 pandemic on tuberculosis treatment outcomes in 49 high burden countries
Source: BMC Med. 2024 Jul 29;22:312. doi: 10.1186/s12916-024-03532-7 (PMC11288071; doi:10.1186/s12916-024-03532-7)
Supplement: Supplementary file 1 — Additional file 1: Figures S1-S3 and Tables S1-S14. Fig. S1 – Country specific observed and expected tuberculosis treatment probabilities in 2021. Fig. S2 – Meta-analyses forest plots for successes and failures in 2021. Fig. S3 – Meta analyses forest plots for deaths and losses to follow up in 2021. Table S1 – WHO region specific observed and expected tuberculosis treatment outcomes in 2020. Table S2 – WHO region specific observed and expected tuberculosis treatment outcomes in 2021. Table S3 – Country specific observed and expected tuberculosis treatment outcomes for the African region in 2020. Table S4 – Country specific observed and expected tuberculosis treatment outcomes for the European region in 2020. Table S5 – Country specific observed and expected tuberculosis treatment outcomes for the South-East Asia region in 2020. Table S6 – Country specific observed and expected tuberculosis treatment outcomes for the region of the Americas in 2020. Table S7 – Country specific observed and expected tuberculosis treatment outcomes for the Western Pacific region in 2020. Table S8 – Country specific observed and expected tuberculosis treatment outcomes for the Eastern Mediterranean region in 2020. Table S9 – Country specific observed and expected tuberculosis treatment outcomes for the African region in 2021. Table S10 – Country specific observed and expected tuberculosis treatment outcomes for the European region in 2021. Table S11 – Country specific observed and expected tuberculosis treatment outcomes for the South-East Asia region in 2021. Table S12 – Country specific observed and expected tuberculosis treatment outcomes for the region of the Americas in 2021. Table S13 – Country specific observed and expected tuberculosis treatment outcomes for the Western Pacific region in 2021. Table S14 – Country specific observed and expected tuberculosis treatment outcomes for the Eastern Mediterranean region in 2021. [file 12916_2024_3532_MOESM1_ESM.docx]

# **Figures, tables and additional files**


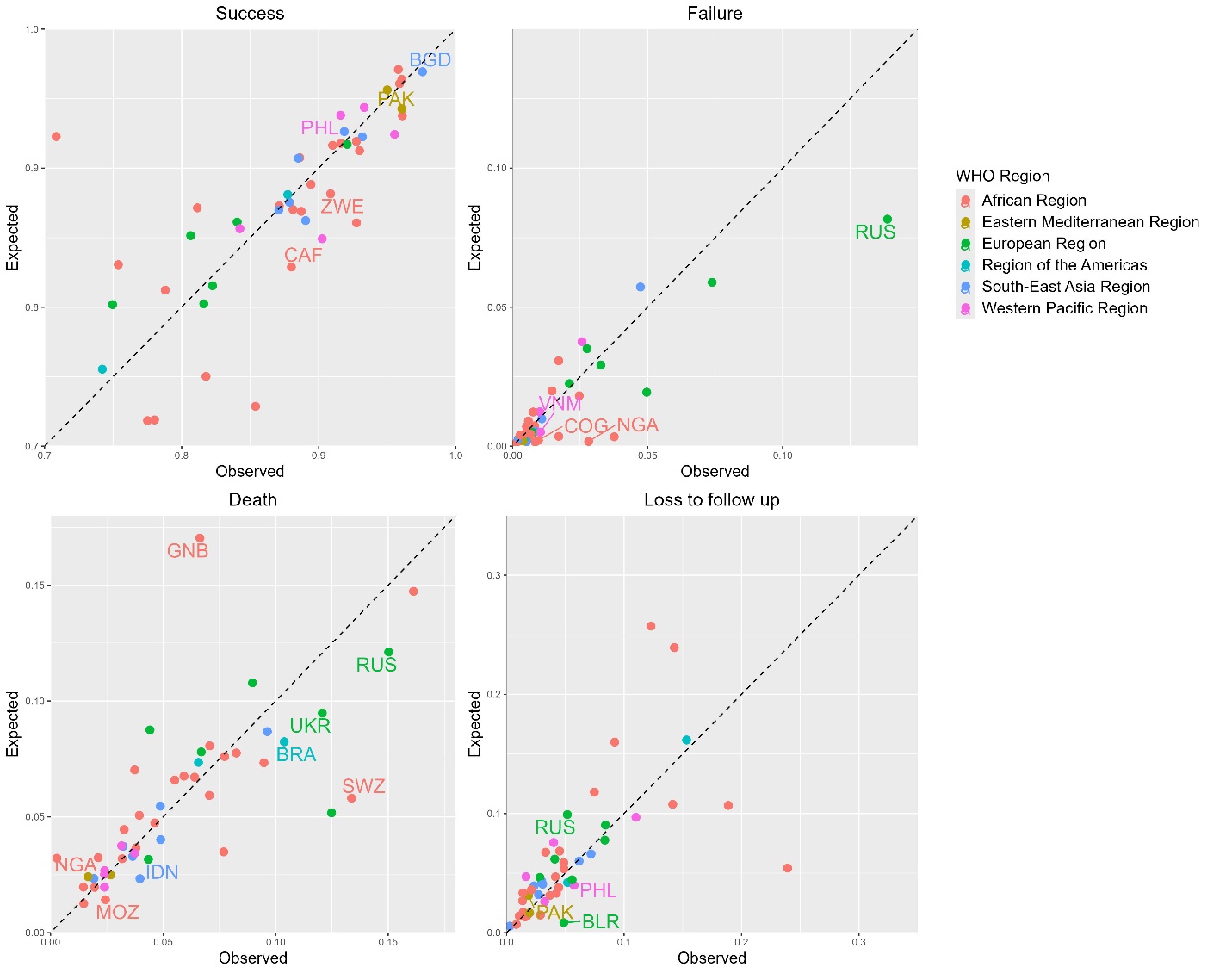


Fig. S1: Observed and expected tuberculosis treatment outcome probabilities for 47 high TB, TB/HIV and MDR/RR-TB burden countries (Kazakhstan and Uzbekistan did not have data for 2021) in 2021. Labelled are those with statistically significant differences: BGD – Bangladesh, BLR – Belarus, BRA – Brazil, CAF – Central African Republic, COG – Congo, GNB – Guinea-Bissau, IDN – Indonesia, MOZ – Mozambique, NGA – Nigeria, PAK – Pakistan, PHL – Philippines, SWZ- Eswatini, RUS – the Russian Federation, UKR – Ukraine, VNM – Viet Nam and ZWE - Zimbabwe.

Table S1: Observed and expected TB treatment outcomes by WHO regions for 2020. Note: EM – Eastern Mediterranean, LTFU – loss to follow up, SEA – South-East Asia and WP – Western Pacific.

| **Region** | **Observed**  **successes** | **Expected**  **successes** | **Observed**  **failures** | **Expected**  **failures** | **Observed**  **deaths** | **Expected**  **deaths** | **Observed**  **LTFU** | **Expected**  **LTFU** |
| --- | --- | --- | --- | --- | --- | --- | --- | --- |
| Africa | 996131 | 989504 | 6437 | 5836 | 51601 | 50095 | 54518 | 63250 |
| EM | 270569 | 268315 | 1122 | 824 | 6455 | 6516 | 6252 | 8743 |
| Europe | 62567 | 65115 | 6990 | 4766 | 9303 | 7689 | 4014 | 5304 |
| Americas | 66333 | 66874 | 172 | 105 | 7489 | 6705 | 10149 | 10458 |
| SEA | 2132625 | 2147181 | 16069 | 12805 | 105391 | 89543 | 85125 | 89682 |
| WP | 897245 | 888492 | 4342 | 3312 | 23243 | 22733 | 23965 | 34257 |

Table S2: Observed and expected TB treatment outcomes by WHO regions for 2021. Note: EM – Eastern Mediterranean, LTFU – loss to follow up, SEA – South-East Asia and WP – Western Pacific.

| **Region** | **Observed**  **successes** | **Expected**  **successes** | **Observed failures** | **Expected failures** | **Observed deaths** | **Expected deaths** | **Observed LTFU** | **Expected LTFU** |
| --- | --- | --- | --- | --- | --- | --- | --- | --- |
| Africa | 1103425 | 1088207 | 11017 | 6371 | 44531 | 52000 | 51831 | 64225 |
| EM | 333524 | 327683 | 1346 | 642 | 5967 | 8405 | 6528 | 10635 |
| Europe | 46859 | 49188 | 6922 | 4361 | 8529 | 6939 | 3522 | 5345 |
| Americas | 69882 | 70861 | 164 | 110 | 8511 | 7228 | 11566 | 11924 |
| SEA | 2513169 | 2530326 | 16470 | 10758 | 122981 | 102290 | 86961 | 96206 |
| WP | 930515 | 921191 | 4175 | 2672 | 24178 | 24351 | 33353 | 44008 |

Table S3: Observed and expected TB treatment outcomes by country for 24 high TB, TB/HIV and MDR/RR-TB burden countries of the WHO African region for 2020. Note: CAR – Central African Republic, DRC – Democratic Republic of the Congo, LTFU – loss to follow up and URT – United Republic of Tanzania.

| **Country** | **Observed**  **successes** | **Expected**  **successes** | **Observed**  **Failures** | **Expected**  **failures** | **Observed**  **deaths** | **Expected**  **deaths** | **Observed**  **LTFU** | **Expected**  **LTFU** |
| --- | --- | --- | --- | --- | --- | --- | --- | --- |
| Angola | 34139 | 36507 | 1524 | 803 | 2244 | 1810 | 7810 | 6597 |
| Botswana | 2270 | 2281 | 14 | 15 | 214 | 197 | 35 | 40 |
| Cameroon | 18899 | 19024 | 118 | 157 | 1441 | 1475 | 1124 | 926 |
| CAR | 9994 | 9465 | 68 | 36 | 546 | 599 | 805 | 1313 |
| Congo | 8408 | 7535 | 63 | 24 | 189 | 140 | 1410 | 2371 |
| DRC | 188943 | 188891 | 524 | 616 | 4521 | 4668 | 3593 | 3406 |
| Eswatini | 1571 | 1668 | 62 | 6 | 195 | 127 | 57 | 84 |
| Ethiopia | 90115 | 88829 | 209 | 263 | 2024 | 2622 | 1496 | 2130 |
| Gabon | 3064 | 4032 | 88 | 32 | 220 | 143 | 1445 | 610 |
| Guinea | 14223 | 14272 | 302 | 368 | 655 | 604 | 372 | 307 |
| Guinea-Bissau | 1908 | 1802 | 22 | 13 | 242 | 362 | 289 | 284 |
| Kenya | 59734 | 60017 | 403 | 495 | 5065 | 4582 | 3642 | 3750 |
| Lesotho | 3390 | 3494 | 28 | 30 | 683 | 639 | 216 | 154 |
| Liberia | 5290 | 5082 | 16 | 36 | 305 | 268 | 1207 | 1433 |
| Malawi | 13320 | 13327 | 49 | 109 | 1094 | 1011 | 237 | 252 |
| Mozambique | 86417 | 86831 | 223 | 176 | 2120 | 1774 | 1408 | 1387 |
| Namibia | 5567 | 5476 | 85 | 123 | 464 | 474 | 180 | 223 |
| Nigeria | 121778 | 121261 | 519 | 337 | 4723 | 4929 | 6308 | 6801 |
| Sierra Leone | 13914 | 13462 | 62 | 43 | 709 | 924 | 535 | 791 |
| South Africa | 130318 | 125139 | 1368 | 1516 | 13437 | 12102 | 17187 | 23553 |
| Uganda | 51617 | 50480 | 339 | 331 | 4326 | 4291 | 3183 | 4363 |
| URT | 80709 | 80615 | 75 | 48 | 2766 | 2999 | 780 | 668 |
| Zambia | 36380 | 36050 | 174 | 163 | 2002 | 1985 | 899 | 1256 |
| Zimbabwe | 14163 | 13964 | 102 | 96 | 1416 | 1370 | 300 | 551 |

Table S4: Observed and expected TB treatment outcomes by country for 9 high TB, TB/HIV and MDR/RR-TB burden countries of the WHO European region for 2020. Note: LTFU – loss to follow up, RF – Russian Federation and RM – Republic of Moldova.

| **Country** | **Observed**  **successes** | **Expected**  **successes** | **Observed**  **failures** | **Expected**  **failures** | **Observed**  **deaths** | **Expected**  **deaths** | **Observed**  **LTFU** | **Expected**  **LTFU** |
| --- | --- | --- | --- | --- | --- | --- | --- | --- |
| Azerbaijan | 973 | 982 | 51 | 31 | 62 | 68 | 86 | 91 |
| Belarus | 710 | 723 | 10 | 19 | 84 | 75 | 23 | 10 |
| Kazakhstan | 5043 | 5205 | 171 | 105 | 461 | 328 | 54 | 91 |
| Kyrgyzstan | 2839 | 2799 | 92 | 90 | 271 | 247 | 254 | 320 |
| RM | 1117 | 1183 | 53 | 45 | 186 | 86 | 33 | 75 |
| RF | 28677 | 30886 | 5570 | 3350 | 6205 | 5153 | 2543 | 3606 |
| Tajikistan | 3332 | 3340 | 44 | 26 | 173 | 124 | 97 | 157 |
| Ukraine | 10232 | 10500 | 852 | 812 | 1377 | 1256 | 744 | 636 |
| Uzbekistan | 9644 | 9497 | 147 | 288 | 484 | 352 | 180 | 318 |

Table S5: Observed and expected TB treatment outcomes by country for 7 high TB, TB/HIV and MDR/RR-TB burden countries of the WHO South-East Asia region for 2020. Note: DPRK – Democratic People’s Republic of Korea and LTFU – loss to follow up.

| **Country** | **Observed**  **successes** | **Expected**  **successes** | **Observed**  **failures** | **Expected**  **failures** | **Observed**  **deaths** | **Expected**  **deaths** | **Observed**  **losses**  **LTFU** | **Expected**  **losses**  **LTFU** |
| --- | --- | --- | --- | --- | --- | --- | --- | --- |
| Bangladesh | 218114 | 218373 | 693 | 529 | 6482 | 5915 | 838 | 1310 |
| DPRK | 77972 | 75897 | 4644 | 5527 | 2874 | 3324 | 3237 | 3978 |
| India | 1364874 | 1374589 | 8235 | 4286 | 70275 | 60164 | 48072 | 52417 |
| Indonesia | 312042 | 318571 | 1205 | 1317 | 13685 | 8397 | 24649 | 23296 |
| Myanmar | 63617 | 63340 | 832 | 721 | 3931 | 3812 | 3690 | 4198 |
| Nepal | 24571 | 24304 | 184 | 162 | 882 | 859 | 605 | 917 |
| Thailand | 71435 | 72107 | 276 | 263 | 7262 | 7072 | 4034 | 3566 |

Table S6: Observed and expected TB treatment outcomes by country for 2 high TB, TB/HIV and MDR/RR-TB burden countries of the WHO region of the Americas for 2020. Note: LTFU – loss to follow up.

| **Country** | **Observed**  **successes** | **Expected**  **successes** | **Observed**  **failures** | **Expected**  **failures** | **Observed**  **deaths** | **Expected**  **deaths** | **Observed**  **LTFU** | **Expected**  **LTFU** |
| --- | --- | --- | --- | --- | --- | --- | --- | --- |
| Brazil | 48548 | 49123 | 46 | 39 | 6137 | 5359 | 9278 | 9488 |
| Peru | 17785 | 17751 | 126 | 66 | 1352 | 1346 | 871 | 970 |

Table S7: Observed and expected TB treatment outcomes by country for 5 high TB, TB/HIV and MDR/RR-TB burden countries of the WHO Western Pacific region for 2020. Note: LTFU – loss to follow up and PNG - Papua New Guinea.

| **Country** | **Observed**  **successes** | **Expected**  **successes** | **Observed**  **failures** | **Expected**  **failures** | **Observed**  **deaths** | **Expected**  **deaths** | **Observed**  **LTFU** | **Expected**  **LTFU** |
| --- | --- | --- | --- | --- | --- | --- | --- | --- |
| China | 576286 | 567281 | 3009 | 1783 | 13970 | 14774 | 10409 | 19836 |
| Mongolia | 3333 | 3203 | 63 | 124 | 129 | 122 | 155 | 231 |
| PNG | 19962 | 20640 | 197 | 292 | 902 | 874 | 3603 | 2857 |
| Philippines | 206635 | 206550 | 612 | 618 | 5855 | 4533 | 7322 | 8723 |
| Viet Nam | 91029 | 90818 | 461 | 495 | 2387 | 2430 | 2476 | 2610 |

Table S8: Observed and expected TB treatment outcomes by country for 2 high TB, TB/HIV and MDR/RR-TB burden countries of the WHO Eastern Mediterranean region for 2020. Note: LTFU – loss to follow up.

| **Country** | **Observed**  **successes** | **Expected**  **successes** | **Observed**  **failures** | **Expected**  **failures** | **Observed**  **deaths** | **Expected**  **deaths** | **Observed**  **LTFU** | **Expected**  **LTFU** |
| --- | --- | --- | --- | --- | --- | --- | --- | --- |
| Pakistan | 255447 | 253312 | 1058 | 775 | 6024 | 6062 | 6084 | 8464 |
| Somalia | 15122 | 15003 | 64 | 49 | 431 | 454 | 168 | 279 |

Table S9: Observed and expected TB treatment outcomes by country for 24 high TB, TB/HIV and MDR/RR-TB burden countries of the WHO African region for 2021. Note: CAR – Central African Republic, DRC – Democratic Republic of the Congo, LTFU – loss to follow up, and URT – United Republic of Tanzania.

| **Country** | **Observed**  **successes** | **Expected**  **successes** | **Observed**  **failures** | **Expected**  **failures** | **Observed**  **deaths** | **Expected**  **deaths** | **Observed**  **LTFU** | **Expected**  **LTFU** |
| --- | --- | --- | --- | --- | --- | --- | --- | --- |
| Angola | 29784 | 32816 | 975 | 716 | 1292 | 1759 | 7460 | 4221 |
| Botswana | 2141 | 2193 | 20 | 13 | 229 | 177 | 26 | 34 |
| Cameroon | 19685 | 19550 | 130 | 176 | 1215 | 1451 | 979 | 832 |
| CAR | 10913 | 10279 | 71 | 30 | 489 | 628 | 927 | 1463 |
| Congo | 9315 | 7948 | 91 | 16 | 161 | 137 | 1341 | 2806 |
| DRC | 204357 | 205046 | 557 | 558 | 4158 | 4165 | 3658 | 2962 |
| Eswatini | 1438 | 1544 | 17 | 4 | 237 | 103 | 80 | 121 |
| Ethiopia | 88480 | 86327 | 383 | 292 | 1949 | 2980 | 1252 | 2465 |
| Gabon | 1845 | 2403 | 98 | 9 | 38 | 51 | 623 | 141 |
| Guinea | 16738 | 16768 | 312 | 561 | 694 | 669 | 526 | 271 |
| Guinea-Bissau | 1858 | 1722 | 41 | 8 | 159 | 408 | 339 | 258 |
| Kenya | 65534 | 64718 | 435 | 673 | 4758 | 4992 | 3640 | 3984 |
| Lesotho | 3470 | 3576 | 36 | 32 | 710 | 649 | 187 | 146 |
| Liberia | 71 | 65 | 0 | 1 | 7 | 3 | 13 | 22 |
| Malawi | 12797 | 12885 | 72 | 100 | 992 | 833 | 199 | 242 |
| Mozambique | 90897 | 92116 | 145 | 142 | 2314 | 1353 | 1514 | 1259 |
| Namibia | 5611 | 5621 | 94 | 128 | 498 | 489 | 237 | 201 |
| Nigeria | 186022 | 184363 | 5646 | 338 | 563 | 6450 | 8336 | 9416 |
| Sierra Leone | 16083 | 14922 | 31 | 28 | 648 | 1218 | 576 | 1170 |
| South Africa | 127431 | 116889 | 1186 | 1914 | 12861 | 12085 | 14340 | 24930 |
| Uganda | 65434 | 64082 | 354 | 336 | 4369 | 4983 | 3590 | 4346 |
| URT | 82496 | 82645 | 74 | 28 | 2736 | 2746 | 712 | 599 |
| Zambia | 46161 | 45308 | 139 | 197 | 2298 | 2352 | 1048 | 1789 |
| Zimbabwe | 14864 | 14421 | 110 | 71 | 1156 | 1319 | 228 | 547 |

Table S10: Observed and expected TB treatment outcomes by country for 7 high TB, TB/HIV and MDR/RR-TB burden countries (Kazakhstan and Uzbekistan had not 2021 data) of the WHO European region for 2021. Note: LTFU – loss to follow up, RF – Russian Federation and RM – Republic of Moldova.

| **Country** | **Observed**  **successes** | **Expected**  **successes** | **Observed**  **failures** | **Expected**  **failures** | **Observed**  **deaths** | **Expected**  **deaths** | **Observed**  **LTFU** | **Expected**  **LTFU** |
| --- | --- | --- | --- | --- | --- | --- | --- | --- |
| Azerbaijan | 1043 | 1034 | 63 | 25 | 56 | 111 | 106 | 98 |
| Belarus | 759 | 778 | 19 | 20 | 81 | 97 | 44 | 8 |
| Kyrgyzstan | 3121 | 3069 | 125 | 112 | 256 | 298 | 322 | 345 |
| RM | 1318 | 1391 | 45 | 57 | 204 | 85 | 67 | 101 |
| RF | 26516 | 28077 | 5583 | 3284 | 6042 | 4873 | 2080 | 3987 |
| Tajikistan | 3347 | 3334 | 27 | 18 | 158 | 115 | 103 | 169 |
| Ukraine | 10755 | 11505 | 1060 | 845 | 1732 | 1360 | 800 | 637 |

Table S11: Observed and expected TB treatment outcomes by country for 7 high TB, TB/HIV and MDR/RR-TB burden countries of the WHO South-East Asia region for 2021. Note: DPRK – Democratic People’s Republic of Korea and LTFU – loss to follow up.

| **Country** | **Observed**  **successes** | **Expected**  **successes** | **Observed**  **failures** | **Expected**  **failures** | **Observed**  **deaths** | **Expected**  **deaths** | **Observed**  **LTFU** | **Expected**  **LTFU** |
| --- | --- | --- | --- | --- | --- | --- | --- | --- |
| Bangladesh | 296487 | 294530 | 684 | 563 | 5862 | 7100 | 819 | 1658 |
| DPRK | 76972 | 74538 | 4092 | 4949 | 2788 | 3223 | 2587 | 3729 |
| India | 1653637 | 1667312 | 9333 | 2928 | 88028 | 72297 | 48967 | 57429 |
| Indonesia | 338738 | 347164 | 1241 | 1309 | 15196 | 8920 | 27518 | 25300 |
| Myanmar | 60939 | 60716 | 750 | 682 | 3384 | 3788 | 4282 | 4168 |
| Nepal | 25631 | 25371 | 227 | 147 | 1003 | 906 | 644 | 1081 |
| Thailand | 60765 | 60695 | 143 | 180 | 6720 | 6056 | 2144 | 2841 |

Table S12: Observed and expected TB treatment outcomes by country for 2 high TB, TB/HIV and MDR-TB burden countries of the WHO region of the Americas for 2021. Note: LTFU – loss to follow up.

| **Country** | **Observed**  **successes** | **Expected**  **successes** | **Observed**  **failures** | **Expected**  **failures** | **Observed**  **deaths** | **Expected**  **deaths** | **Observed**  **LTFU** | **Expected**  **LTFU** |
| --- | --- | --- | --- | --- | --- | --- | --- | --- |
| Brazil | 50439 | 51339 | 55 | 34 | 7055 | 5600 | 10415 | 10991 |
| Peru | 19443 | 19522 | 109 | 76 | 1456 | 1628 | 1151 | 933 |

Table S13: Observed and expected TB treatment outcomes by country for 5 high TB, TB/HIV and MDR-TB burden countries of the WHO Western Pacific region for 2021. Note: LTFU – loss to follow up and Papua New Guinea.

| **Country** | **Observed**  **successes** | **Expected**  **successes** | **Observed**  **failures** | **Expected**  **failures** | **Observed**  **deaths** | **Expected**  **deaths** | **Observed**  **LTFU** | **Expected**  **LTFU** |
| --- | --- | --- | --- | --- | --- | --- | --- | --- |
| China | 537587 | 520067 | 2255 | 1057 | 13527 | 15085 | 9314 | 26474 |
| Mongolia | 2317 | 2180 | 66 | 97 | 81 | 96 | 103 | 194 |
| PNG | 20705 | 21049 | 247 | 304 | 918 | 844 | 2706 | 2380 |
| Philippines | 299789 | 306999 | 831 | 831 | 7850 | 6430 | 18797 | 13007 |
| Viet Nam | 70117 | 70896 | 776 | 383 | 1802 | 1896 | 2433 | 1953 |

Table S14: Observed and expected TB treatment outcomes by country for 2 high TB, TB/HIV and MDR/RR-TB burden countries of the WHO Eastern Mediterranean region for 2021. Note: LTFU – loss to follow up.

| **Country** | **Observed**  **successes** | **Expected**  **successes** | **Observed**  **failures** | **Expected**  **failures** | **Observed**  **deaths** | **Expected**  **deaths** | **Observed**  **LTFU** | **Expected**  **LTFU** |
| --- | --- | --- | --- | --- | --- | --- | --- | --- |
| Pakistan | 317293 | 311345 | 1283 | 605 | 5511 | 7979 | 6193 | 10351 |
| Somalia | 16231 | 16338 | 63 | 37 | 456 | 426 | 335 | 284 |


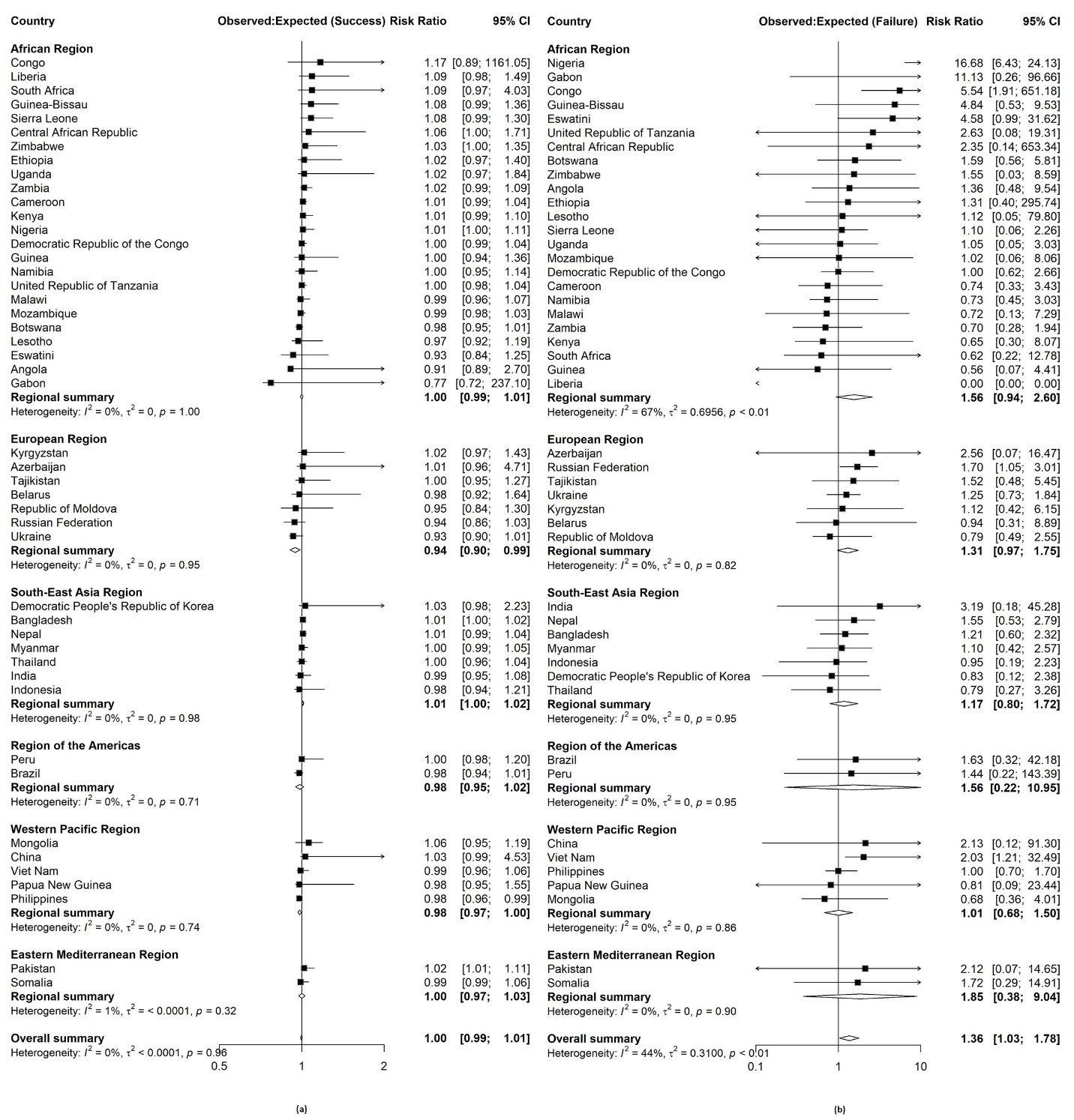


Fig. S2: Random effect meta-analyses forest plots highlighting ratios between observed and expected proportions for tuberculosis treatment success (a) and failure (b) in 2021 for 47 high TB, TB/HIV and drug resistant TB burden countries (Kazakhstan and Uzbekistan did not have data for 2021) by WHO region. Greater than one ratio imply that the observed proportions were more than expected, and less than one ratio imply that the observed proportions were less than the expected.


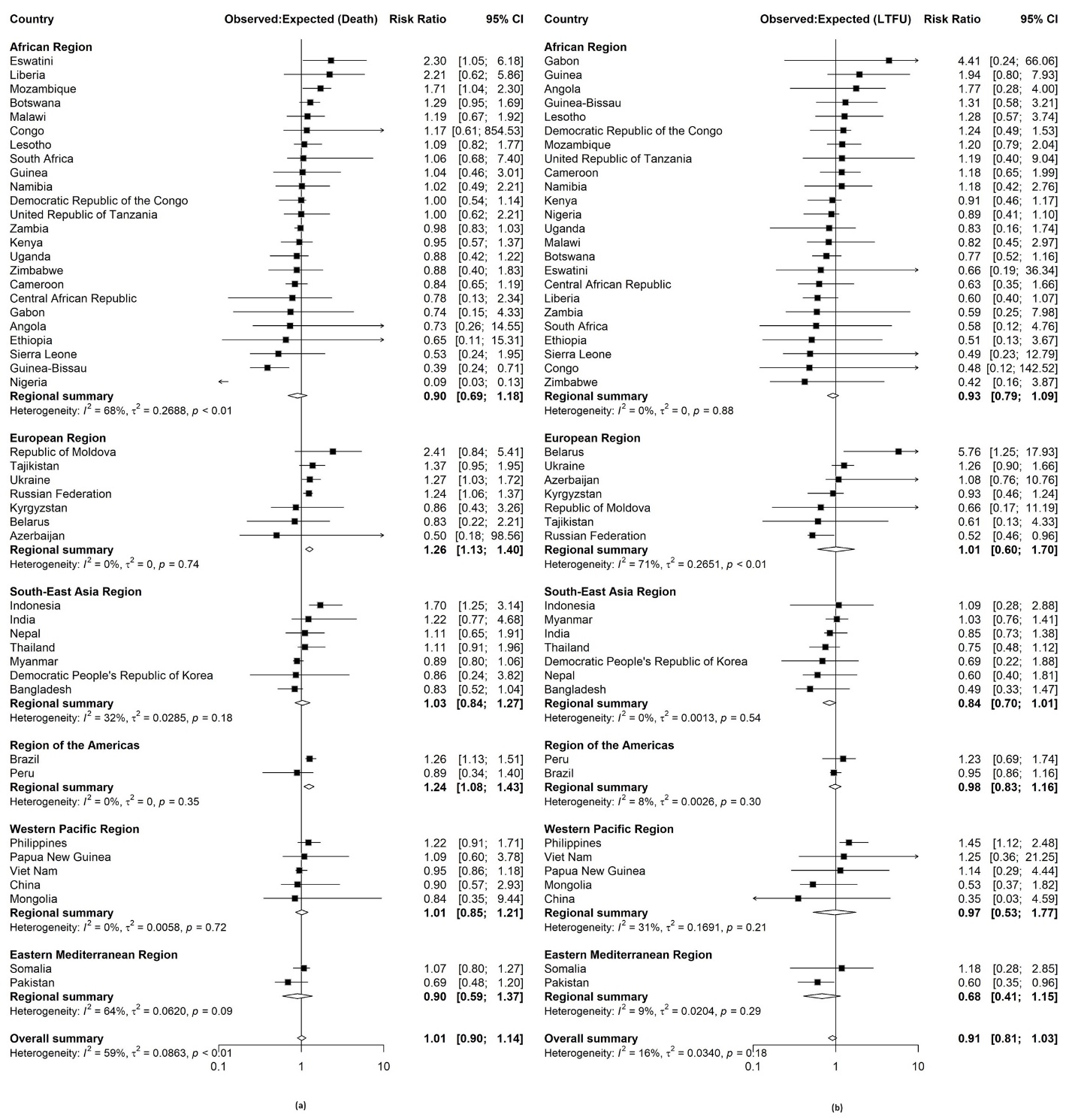


Fig. S3: Random effect meta-analyses forest plots highlighting ratios between observed and expected proportions for tuberculosis treatment death (a) and loss to follow up (LTFU) (b) in 2021 for 47 high TB, TB/HIV and drug resistant TB burden countries (Kazakhstan and Uzbekistan did not have data for 2021) by WHO region. Greater than one ratio imply that the observed proportions were more than expected, and less than one ratio imply that the observed proportions were less than the expected.
